# False Issues and Fanciful Facts
*"Dying Scientifically: A Key to St. Bernard's." By Æsculapius Scalpel. London: Swan Sonnenschein, Lowrey, and Co. 1888.


**Published:** 1888-08-25

**Authors:** 


### False Issues and Fanciful Facts.*

[Books for Review should be sent to The Editor, The Lodge, Porchester Square, W.]

The sensational title of this *brochure* has been adopted to head a collection of extracts from the medical journals designed to justify the publication of "St. Bernards," by its author. No more serious blow could have been dealt to our existing Hospital System than this book, could its statements and innuendos be verified by positive evidence. The author claims to have established 75 per cent. of what appears there as positive fact, while the remaining 25 per cent. he is pleased to call romance—an euphonious description of the matter in question, to which many of his readers would be inclined to apply a less-attractive term. One instance of what Dr. Scalpel views as romance may be quoted without comment. Under the heading of "Count your Sponges, Sister," the following publicly-reported case is romanticised:—

British Medical Journal.

"In a second case, E. R., aged 39, a tedious and difficult operation, I placed a drainage tube, as the tumour had been so adherent as to require a partial enucleation. Six hours after the operation the sister at the hospital telephoned me that they missed a torsion forceps. *On seeing the patient at once*, I found her fairly well, though in pain, and I recommended a further search. Next morning, exactly twenty-four hours after the operation, she was in intense pain, anxious, quick pulse, though the temperature was not much raised; but as the forceps could not be found, I concluded that it was in the patient's abdomen, and under the spray I cut the uppermost stitch and made from it a fresh opening into the abdomen above the umbilicus. After a short search, I found the forceps with the lymph upon it, lying down against the left side of the spine. I made the fresh opening so as not to interfere with the drainage tube, or to allow its contents to enter the abdomen. The patient made a perfect recovery."

Æsculapius Scalpel.

"The telephone bell rang: Dr. Stanforth rose from the dinner table, put the instrument to his ear, and asked who wanted him. 'Sister Agnes, Doctor! I have lost a pair of *dressing* forceps; have you taken them away by mistake?' Dr. Stanforth feels in his pockets, looks in his little black bag, which was lying on the sideboard, and fails to find them. He rings the telephone bell and informs the sister he has not taken them, and has not the least idea where they are. *Wishes her good evening and returns to his dinner.* Next morning he goes his rounds and pays an early visit to the woman who, twenty-four hours ago, underwent at his hands the terrible operation of ovariotomy. Sister Agnes reproachfully remarks that she had not found *her* forceps. The poor patient had complained to him of a terrible pain in the back. 'Happy thought!' cries Stanforth, slapping his knee, 'shouldn't wonder if I haven't sewn 'em up in her abdomen! Let's have a search!' The sufferer is anæsthetised, imploring the doctor not to cut her about any more. The stitches are undone, another incision is made, to facilitate the search, and lo! the missing instrument is found lying snugly under the left kidney."

Our Medical Students.

The original work was directed not against the hospital system alone, but took cognisance also of the whole question of student life and medical education, and it is for justification of his general statements on these matters that we are provided with the book before us. In "St. Bernard's" the medical student is held out to us in his most unattractive light; every bad attribute which could be seized upon is compressed into a few characters, while, with the exception of his ideal Elsworth, no single instance of goodheartedness, earnestness of work or purpose, are met with. In support of this general character some quotations from the daily press are made, relating to disturbances in two provincial towns, attributed to the students belonging to the local schools, and a short extract regarding the possible evasion of lectures by students. In spite of the author's disclaimer, we think that few of those acquainted with the student of to-day will deny that his description is highly coloured, and more in accordance with the romance of Dickens than the actual state of affairs. The medical student is, no doubt, under considerable social disadvantages. Introduced at a comparatively tender age to all the temptations and dangers of a practically free and uncontrolled life in London or elsewhere, it is not surprising that a certain percentage do fall victims to the many snares surrounding them, but that the average student in any way corresponds to the description in "St. Bernard's" is untrue, and in no way supported by evidence in "Dying Scientifically." As to attendance at lectures, as Dr. Scalpel must well know, the size of the class depends entirely upon the matter provided by the lecturer, while in any case a certain percentage of the lectures must be attended, to obtain the signatures to the schedule necessary for presentation prior to examination. When the large number of students in London (more than forty-eight per cent. of those studying in the United Kingdom) is taken into consideration, it would be difficult to prove that they are more inclined to rowdyism than any other body of young men similarly situated. A proof of this assertion indeed exists in the reports occasionally coming to light of the behaviour of students at the Universities of Oxford and Cambridge, where not only are the men under strict tutorial charge, but such disturbances as do occur are for the most part adjudicated upon by the University authorities, instead of by the police magistrate, as is the case in London. The responsibility of such rowdyism as does occur is laid by the author of "St. Bernard's" on the heads of the hospital authorities. Anyone acquainted with the teaching staff of the large London hospitals will know that as far as lies in their power, considerable social attention is bestowed on the students by them; and if the public in general would emulate their efforts by showing a moderate amount of social civility to such students as they may be acquainted with, many a young fellow who now from a mere sense of isolation in a great city falls into evil ways, might never even give a shadow of support to such allegations as are made in the romance of "St. Bernard's."

Slightly Inconsistent.

A similar series of grumbles at the mode of education at present in vogue follows, but while on the one hand the fact that the general hospitals are open to students for the acquisition of knowledge is criticised, the author, with singular inconsistency, demands their compulsory attendance at hospitals for infectious disease, the insane, &c. Apart from the difficulty of carrying out the attendance at fever hospitals, and the disadvantage of the free entry of students into asylums, the chief aim and object of which is to keep the patients as quiet and free from any source of excitement as possible, it is well known that the only way in which such special knowledge could be thoroughly attained would be the lengthening of the professional curriculum, already as prolonged as the pockets of the great majority of parents can tolerate. In spite, then, of the moderate fees at present paid by our students, this no doubt very desirable change could hardly be made, and with regard to the lament over the advantages of the now almost obsolete system of apprenticeship (by which medical men in the author's sphere of practice derived a considerable income) most people would agree that such experience as was gained by a schoolboy during his pupilage with a country practitioner is at any rate as well acquired by the young qualified assistant or partner subsequent to obtaining his diploma. This, of course, cannot

* "Dying Scientifically: A Key to St. Bernard's." By Æsculapius Scalpel. London: Swan Sonnenschein, Lowrey, and Co. 1888.

apply to the young practitioners who are at once launched on their own resources; but even in regard to them, we think that anyone who can compare the average general practitioner of to-day with his predecessor of thirty years ago, under the old system, will allow that in actual knowledge of his profession the advantage remains with the younger generation.

No Experiments.

As to the onslaught on the methods of observation and treatment, it is difficult to speak critically, for our author, while showing enough information to allow of his identification as one who has medical knowledge, exhibits utter indifference and aversion to progressive medical and surgical treatment, as well as ignorance of its present degree of advancement. In "St. Bernard's," we are told that at the Nightingale Hospital (the author's ideal) no new operations or drugs, unless well established elsewhere, were permitted by the governing body. It stands to reason that were this a general rule, the whole science would come to a standstill, a consummation, in spite of the good intentions of Dr. Scalpel, which would scarcely meet with wide appreciation. The examination of cases by students so warmly animadverted on is supported by the reports of some cases of brain disease and functional disorder (a patient with hysterical catalepsy is "romantically" described as a "dying creature" in this work of scientific proof) the careful and systematic observation of which is regarded not as having helped to build up the grand structure of cerebral localization and therapeutics gradually being involved with not only theoretical, but practical benefit to the whole world, but as observation "necessary to make a case for the journals." As an example of the character of observasion preferred by Dr. Scalpel, we may quote a case spoken of by him in support of his description of sudden cures. "A lady afflicted with 'a disease of the knee,' whose cure was as sudden as the attack. A serious operation seemed to demand her presence; she jumped out of bed and resumed her duties as if nothing had been the matter." As to treatment by drugs, the result of the perusal of the extracts furnished is somewhat mystifying. In some instances of Sir A. Cooper's condemnation of medical treatment in general, and in the report of a case of exophthalmic goitre, which improved without the exhibition of any drug, the author seems to disapprove of any medicinal treatment, while in others, as the report of some cases of typhoid fever quoted, he appears to condemn the absence of such procedure. The experiments on the administration of alcohol and nitrite of soda have already been brought before the public, who then had an opportunity of expressing an opinion on the subject, and while some may be inclined to disagree with the way in which these were carried on, yet it must be borne in mind that every drug which we have at command has been originally administered in ignorance of its therapeutic properties, and thus, as is the case with every new operation, its employment must have been in some sense an experiment.

Withholding the Truth.

On this topic we may point out that although these efforts are made by an anonymous writer, to discredit members of his own profession by name, no mention is made of a large number of similar experiments which have been made by noted therapeutists on themselves or their pupils; or again, while the cruelty of skin grafting is dilated upon, does the student who gives his own skin, or another who supplies his own blood for transfusion into a hospital patient come into notice, in contrast to the youths in "St. Bernard's," who seem to take a wicked delight in torturing the unfortunate patients who come under their charge. With regard to a large number of operations assailed, such as trephining, operations on large joints and arteries, we may merely notice that these are some of those to which modern surgery can point with legitimate pride as constituting great and admirable advances. Again, what answer can be expected to a medical man who, in condemning the treatment of aneurisms of the chest by the introduction of steel wire, makes the remark of the surgeon that the right quantity of wire is still undetermined, a peg on which to hang the following commentary, "What, not after all the dogs, guinea pigs, and rabbits that were to set these matters to rights!" It would seem that even a school-girl with some notion of what a large thoracic aneurism is, would scarcely be suspected of looking for information in the direction which suggests itself to Dr. Scalpel. The performance of novel and major operations by house-surgeons on their own responsibility is retracted, but an attempt to give some colour to the assertions is made by the quotation of a single instance, and also the account of a case in which the house surgeon punctured the brain of a patient who had been trephined and evacuated some pus, with the result, apparently, of prolonging the patient's life for a week. The whole matter, however, is treated in a disengenuous way: we are told that the exigences of the romantic style is accountable for the substitution of the house-surgeon for the assistant surgeon. The author, probably again trusting to the ignorance of his readers, placing the latter officer at as low a point in professional standing as the prefix could imply, while no reference is thought necessary to the more startling and romantic offer to a pupil by Dr. Stanforth of the use of his patients for the practice in the performance of such operations as gastrostomy.

Operating on the Dying.

In support of the allegation that operations are needlessly performed on dying patients, some statistics antagonistic to the operation of tracheotomy by a supporter of the treatment by intubation, are offered, instead of such results as might readily have been attained by an examination of the annual hospital reports, while a report of an unsuccessful abdominal section for gunshot wound, a mode of treatment more than justified by the brilliant results recently obtained in cases of ruptured intestine and bladder, needs no further remark. As a commentary on another allegation that new operations are reserved for the ignorant and helpless hospital patient, the case of successful removal of a tumour from the spinal cord, so recently before the public, may be cited as an instance of a new operation done on a private patient, while the examination of the results of any of the principal operators of the day would at once show that a large number of operations of the nature condemned by Dr. Scalpel are performed on persons of the upper classes. As to Dr. Scalpel's ideal of a medical man, we are left in the dark; and in "St. Bernard's," with the exception of Elsworth, the only words of commendation we find are for Dr. Stanforth, who, though portrayed as a liar, a fool, and in every sense the opposite of a gentleman, we are told was "everything that could be required in a doctor to his patients." A general estimate of the character of our leading physicians and surgeons can, however, we feel, be safely left to those who come under their care, for, in spite of the criticisms of work in a special department quoted in "Dying Scientifically," there is little doubt that if the bad points of all the medical staff of a large hospital were compressed into one individual, he would make but a feeble figure by the side of such characters as Dr. Scalpel's imagination has evolved.

Cui Bono?

It is difficult to see what use these extracts can serve, except in showing to the public that what is done in our hospitals is not the dark secret that might be inferred by an uninitiated reader, into whose hands "St. Bernard's" might fall; but that, on the contrary, such new treatment as is resorted to is fully ventilated to a profession capable of examining critically, and of giving an outspoken opinion on any new procedure; and, again, that successes, accidents, and failures are published for the common good. It may be well to emphasize here also the further fact that the medical journals are not read and criticised by the profession alone, and an article by our author in another place fully bears out this statement.

With one remark we may close this lengthy notice. Albeit bringing grave charges against his compeers and the *alma mater* he professes to love, Dr. Scalpel, in "Dying Scientifically," prefers to remain anonymous, although mentioning many prominent physicians and surgeons by name; and while in "St. Bernard's" the minute description of many details, and particularly the tragic end of one of the romanticised professors, leaves no doubt of the institution he purports to describe. Whether this bears out the reasons which he gives for still withholding his name we must leave his readers to decide.

